# Observations on the Internal Use of Nitrate of Silver

**Published:** 1818-06

**Authors:** William Balfour


### *Observations on the Internal Use of Nitrate of Silver.*
By William Balfour, M. D.

Case 1. On the 14th of August, 1816, Mr. A. applied to me, oppressed with a variety of very distressing complaints. The leading features of these were, general debility; debility of the inferior extremities, approaching to paralysis; complete relaxation of the sphincter ani; prolapsus of the gut, accompanied with frequent loss of blood; and a perpetual and copious gleety discharge. He dated the commencement of his complaints fourteen years back. Had put himself under the care of some of the most eminent practitioners in this city; from whom he derived no benefit. He afterwards went to London, for the purpose of consulting the late Dr. Beddoes, who guessed the cause of his complaints the moment he saw him walk. Dr. Beddoes asked him if ever he had received an injury in the back? The patient declared positively he never had. But upon the doctor's insisting on it, came at last to the recollection of having been struck forcibly on the lumbar vertebræ by the shaft of a gig, a short time before he began to complain. From Drs. Beddoes and King, the last of whom was likewise consulted, he derived no further benefit than what resulted from the application of ligatures to some vessels of the rectum, by which hæmorrhage was checked to a very considerable degree.

Mr. A. applied to me in the hope of profiting from those means by which I had succeeded in restoring some rheumatic gouty limbs, as detailed in my Treatise on Rheumatism. I began with gentle percussion to the sacrum, the glutei muscles, and the course of the sciatic nerve; with the view of exciting the action of the nerves supplying these parts, and of eliciting a transmission of nervous power from the spinal marrow. This operation was soon followed by increased command and power of the limbs; but had not been repeated above three or four days, when increased discharge of blood from the rectum took place. Till this occurrence, indeed, I was kept in ignorance of the state of the anus, having my attention directed to the imbecility of the limbs solely. I now gave up the idea of percussion as impracticable, and prescribed the most powerful liquid astringents to the bleeding surface, which was quite exposed to view, but with little or no effect. The patient requested me "to take up the veins as Dr. King had done," but I could perceive nothing but an oozing from an extensive surface. I had now recourse to nitrate of silver as an internal astringent, in the quantity of a six teenth of a grain three times a day. I was agreeably surprized when, after two or three days had elapsed, the patient informed me, the sense of fulness which he always perceived to precede a discharge, had totally left him, and that the discharge itself, of blood, was also greatly diminished. In a short time, the bleeding ceased altogether, and the gleety discharge also began to be sensibly diminished. I wished, at this period, to increase the dose of the medicine, but found it impracticable, on account of the excessive perspiration it occasioned—an effect this, which was not, *a priori*, to be expected. But even with the minute quantity of a sixteenth of a grain three times a day, sometimes only twice a day, and sometimes omitted altogether, the gleety discharge had, in two months, ceased almost entirely, the sphincter resumed its functions, and the anus contracted and puckered in the natural way.

Such mighty effects may be considered by the reader as out of all proportion to a cause apparently so trifling; unless he is disposed with me, to infer, that the nitrate of silver, as an internal remedy, has hitherto been overlooked, and its powers under-rated. It cannot be supposed, that three sixteenths of a grain of nitrate of silver could come in contact with the whole surface of the intestinal canal. Had it even been applied directly to the bleeding surface, I am convinced it would have had no beneficial effect. The effects produced must, therefore, have been through the system.

Case 2. Helen Thomson, aged 32, of a cadaverous countenance, and whose cousins and sisters all died of consumption, came under my care on the 9th of January, 1817. She complained of great general debility; of profuse perspiration on the slightest exertion; of frequent giddiness, especially on turning quickly round; and of a sense of weight in the back part of the head.

The state of the pulse I could not satisfactorily ascertain, as I never saw the patient but after, what was to her, a long walk. From the history she gave me of her family, more than from her present symptoms, I considered this woman gone. I prescribed two dozen of pills, each containing an eighth of a grain of nitrate of silver; one to be taken morning, mid-day, and evening. On the 19th she returned, quite delighted with the beneficial effects of the pills. I now ordered them of one fourth of a grain each; and she continued taking three a day till about the end of February;—in all, eight dozen. She had now recovered her strength; the sweatings were checked; and she could do her work, being a servant, with perfect ease. I saw her again in the street, about the middle of summer, full of flesh, and apparently in perfect health. With the exception of a laxative pill, occasionally, this patient had no other medicine than the nitrate of silver; and she described its invigorating effects as almost instantaneous.

Case 3. Mr. William Elliot, aged 19, came under my care on the 20th of October, 1816. He had been complaining for nine months back, of pain in the breast, attended with cough and dyspnœa, the latter attacking him in paroxysms almost to suffocation. Had had a good deal of medicine from different practitioners, but found himself getting weaker daily. He was now, indeed, very much reduced, and unable to take much exercise in the open air. I was called in to him at first in a great hurry, owing to his being suddenly seized with a most violent stitch under the short ribs, right side. From the state of his pulse, I was no way apprehensive of inflammation, and therefore satisfied myself with the application of percussion for two minutes, by which he felt himself greatly relieved, and was enabled to turn himself any way he pleased. In about a quarter of an hour he became very sick, and vomited a quantity of greenish yellow substance, very offensive to the taste, and was immediately and entirely relieved of all his complaints. I ordered an aperient medicine. On the 4th of November I was again called in a very great hurry, and found the young man labouring under a tremendous fit of asthma, with a frequent, full pulse, and considerable heat of skin. I took fourteen ounces of blood from the arm, with some relief of symptoms. Next day he had another paroxysm of asthma, when I ordered him a grain pill of opium. This had the desired effect, not only at this time, but ever afterwards; nor was it ever necessary to increase the dose of opium. One grain, taken when a paroxysm was threatened, completely checked it. Finding, however, that debility, emaciation, and sweating continued, I began, on the 25th of November, with the nitrate of silver, in the quantity of a sixteenth of a grain twice a day, or, as sweatings occurred. This had the effect of moderating the sweating. On the 9th of December, I increased the dose to an eighth of a grain, to be taken at any time, by day or night, when menaced with profuse perspiration. On the 11th December, the dose was increased to a fourth of a grain. The patient's strength was now evidently improved, and he checked the sweatings at any time by taking one pill; nor was the asthma or cough at all troublesome. He continued the nitrate of silver pill till about the end of June, a rhubarb or aloetic pill being now and then interposed to keep the bowels regular, as the nitrate of silver had rather a constipating effect. Every symptom of disease had now disappeared; the patient had recovered flesh, strength, and a healthy appearance. I considered his recovery complete. About the middle of September, however, he was seized with heartburn, which, in a few hours, was succeeded by vomiting a considerable quantity of a dark coloured substance, so thick and tenacious, that it might have been suspended on a stick. Upon this, the patient was again perfectly well; nor did any of his former symptoms return. I ordered him some laxative medicines, but the same phænomenon recurred repeatedly at short intervals. I had now recourse to the blue pill as an alterative, and with the happiest effects; of these he took five dozen in the course of September and October, when he gave over all medicine. Through the course of the succeding winter, he had few complaints, and used as little medicine. On the approach of spring, however, he was again menaced with a return of heartburn and asthma; symptoms which were immediately checked by emetic tartar, exhibited in the form of pill, and in the quantity of a fourth of a grain at bed-time, as occasion required. If there was any affection of the liver in this case, the blue pill seemed to have a good effect on it; but emetic tartar a better: and I regret much I did not exhibit this latter medicine sooner. I cannot but attribute, however, the first check the original complaints received to the nitrate of silver.

Case 4. Mrs W. a married lady, about 36 years of age, of a fine delicate complexion, and who never had any children, consulted me in May 1817, with regard to fluor albus, under which she then laboured, and to which she had been occasionally subject for some years. I prescribed a lotion, as the acrid nature of the discharge occasioned some uneasiness, and the nitrate of silver pill. She took only two dozen of one-fourth of a grain each, when the discharge disappeared. My patient was very much surprised at the decided effects of the medicine, as she had been, on a former occasion, under the care of an eminent Surgeon for the same complaint, and experienced little relief for a great length of time, although she used a great deal of medicine internally.

Case 5. On the 2d of August 1817, I was consulted by letter, in the case of Mr. Thos. Coutts, Kinross-shire. Five weeks previous to this, Mr. Coutts was seized with cough and spitting of blood, which continued for a week, when he was bled in the arm to the amount of "fourteen or sixteen ounces." In a few days the spitting of blood returned, when a blister to the breast had the effect of again stopping it The patient was extremely weak, and had little or no appetite. Pulse ranged from 80 to 95. I recommended the nitrate of silver, one-fourth of a grain, four times a day. On the 20th I had a return from the patient himself, stating, "I cannot say much yet, only my stomach is better, and my breathing a little more free. I sleep fully as well, and do not sweat in the night so much. What I spit is not so gross; it is more mixed with spittle, or something the appearance of common spittle." On the 10th of September, after complaining of being much harased with cough, he says, "I have taken three pills every day, finding four too many for me; but as they are the best thing that I have got, you may send me another box or two." I now desired the patient to give up the pills for a few days, in order to ascertain if they were of any real benefit. With this advice he complied; and on the 17th informed me, "I tried to want the pills, but it would not do. I did not feel much the first day, but the second I was very bad in my breathing, and could not say how in my inside; so I began again, three in the day." On the 15th of October he requested me to send him another box of pills, adding, "perhaps I may not need any of them; but if I do, (meaning if he lived) I cannot want them.—You will be sure to send it this week." He died in about ten days after.

It is evident, from the patient's own account, that in his case the nitrate of silver obviated the formation of pus, and improved its quality; that it checked the hectic perspiration, and facilitated perspiration by supporting the tone of the system.

Case 6. Mr. S. about 36 years of age, of a strongly marked scrofulous habit, and having had for many years a copious purulent discharge from the lungs, and difficult respiration, was attacked last summer with what was deemed confirmed consumption. After being greatly reduced, and confined for some time to the house, he came abroad again, still discharging immense quantities of purulent-looking matter, of a bad smell and taste. His return to the world was considered by his physician, and with much reason, a temporary respite only. About the middle of

July he consulted me, and I instantly put him upon nitrate of silver, a fourth of a grain four times in the day. Having taken three dozen of pills in this way, he returned for a supply; informing me, that the sputum was reduced in quantity, and entirely deprived of its bad taste and smell; and that he now coughed with a vigour, and expectorated with a freedom, to which he had been long a stranger. In the beginning of August he went to the country, carrying along with him twenty dozen of the pills, which he was directed to take as he found them affect him. He returned to town in the beginning of October, much improved in every respect, and having exhausted his stock of pills. He now took other four dozen, when he dropped them altogether, having no further occasion for medicine.

I will not say, that in this instance nitrate of silver cured phthisis; but from the authority of the patient, and the testimony of my own senses, I affirm, that the quantity of sputum was diminished, and its qualities improved; and that a tone and vigour was communicated to the constitution, which no other medicine with which I am acquainted could have imparted.

Case 7. Mrs. Simpson, aged about 30, of an extremely fine complexion, and delicate frame, consulted me in November last, with regard to her general health, which was much impaired. She exhibited, indeed, the appearance of being far advanced in phthisis. I found she had laboured for some time under fluor albus; and that the discharge was copious, extremely acrid, and accompanied with distressing pain in the region of the uterus. I immediately put her on the nitrate of silver pill, with immediate and great good effect. The pain went off in three days, and the discharge was lessened in proportion. In less than a fortnight she felt her strength considerably improved, and in every respect much amended. She continued the medicine about a month, at the end of which she was perfectly free from complaint. I cannot, indeed, convey an adequate idea of the change, for the better, produced on this interesting and delicate female.

Case 8. Mrs. M. who had been married several years, but had no children, tall, slender, had constantly an eruption all over her face of a fiery red, and who had not menstruated for several years, became subject to an abscess in a particular spot, within the left *labium pudendi,* of frequent recurrence. She underwent three courses of mercury, but the complaint perpetually recurred. At length,

I prescribed nitrate of silver with the happiest effects. Not only did the discharge disappear, and the ulcer heal up kindly, but the whole habit of the patient seemed to undergo a revolution; her face even became less fiery and red, symptoms of the return of the catamenia began to manifest themselves, and she felt herself better than she had been for several years before. It was with some difficulty I could persuade this patient to drop a medicine from which she had experienced so much benefit.

CASE 9. A lady, advanced in life, was seized with a pneumonic affection, accompanied with colliquative perspiration. As soon as the pain of the chest was subdued, which was effected *without blood-letting*, and that the patient could make a full inspiration, nitrate of silver was exhibited. The perspirations were immediately checked, and the strength of the patient was quite restored in a short time.

CASE 10. A young man was attacked with slight, obtuse pain in his chest, for which he took advice, but of what nature I know not. When I was consulted I found him much reduced by colliquative perspiration. This was, indeed, his only complaint: and the nitrate of silver removed it entirely in a very few days.

I have exhibited nitrate of silver in obstinate gleets from gonorrhœa; and in many instances with perfect success, after all the usual remedies had been tried in vain. I have likewise failed in some cases; but believe it was more owing to the medicine not being carried a proper length, than to its inefficacy. In some cases I have made a cure in a very few days; in others, a considerable time elapsed before much effect was produced. One gentleman had been repeatedly under my care for gonorrhœa, which in every instance was difficult of cure. The patient had a cadaverous, unhealthy appearance. The last time he consulted me, I prescribed nitrate of silver, after the inflammatory symptoms were subdued; and with immediate good effect. Not only was the discharge quickly dried up, but the patient described himself as having acquired a tone and vigour to which he was formerly a stranger. It was with difficulty also that this patient was persuaded to give over the medicine.

Such are some of the effects I have observed from the internal use of nitrate of silver—effects which entitle it to more attention than has yet been bestowed on it. From the preceding statements, it is evident, that it possesses anti-purulent powers in no common degree; and, that in depraved and relaxed habits, it is a remedy that has no rival.

W. BALFOUR, M. D.

